# Effects of Zinc Acetate Hydrate Supplementation on Renal Anemia with Hypozincemia in Hemodialysis Patients

**DOI:** 10.3390/toxins14110746

**Published:** 2022-10-31

**Authors:** Eiichi Sato, Shohei Sato, Manaka Degawa, Takao Ono, Hongmei Lu, Daisuke Matsumura, Mayumi Nomura, Noriaki Moriyama, Mayuko Amaha, Tsukasa Nakamura

**Affiliations:** 1 Division of Nephrology, Department of Internal Medicine, Shinmatsudo Central General Hospital, Matsudo 270-0034, Japan; 2 Department of Nephrology, Kashiwa Forest Clinic Kashiwa, Kashiwa 277-0042, Japan

**Keywords:** zinc acetate hydrate, hypozincemia, renal anemia, hemodialysis patients

## Abstract

Introduction and Aims: This study examined whether zinc supplementation with zinc acetate hydrate improved renal anemia with hypozincemia in patients undergoing hemodialysis. Methods: The study participants included 21 patients undergoing hemodialysis who presented with a serum zinc level < 60 mg/dL and who were administered zinc acetate hydrate at 50 mg (reduced to 25 mg, as appropriate) for 6 months. Patients with a hemorrhagic lesion, acute-phase disease (pneumonia or cardiac failure), or hematologic disease and those whose treatment was switched from peritoneal dialysis to hemodialysis were excluded. The changes in the erythropoietin resistance index (ERI) before and after zinc acetate hydrate administration were examined. ERI was defined as the dose (IU) of erythropoiesis-stimulating agent (ESA)/week/body weight (kg)/hemoglobin content (g/dL). The differences between the two groups were analyzed using the Wilcoxon signed rank sum test, and *p* < 0.05 was considered statistically significant. Results: The study participants included 19 men and 2 women aged 41–95 years (mean ± standard deviation (SD): 67.1 ± 13.6). The changes in the values of parameters measured before and after zinc acetate hydrate administration were as follows: Blood Hb did not change significantly, from 10.0–13.6 g/dL (11.5 ± 1.0 g/dL) to 10.2–12.4 g/dL (11.4 ± 0.7 g/dL); serum zinc concentration significantly increased, from 33.0–59.0 mg/dL μg/dL (52.4 ± 7.6 mg/dL μg/dL) to 57.0–124.0 mg/dL μg/dL (84.1 ± 16.3 mg/dL μg/dL; *p* < 0.01); the ESA dose significantly decreased, from 0–12,000 IU/week (5630 ± 3351 IU/week) to 0–9000 IU/week (4428 ± 2779; *p* = 0.04); and ERI significantly decreased, from 0.0–18.2 (8.1 ± 5.1) to 0.0–16.0 (6.3 ± 4.3; *p* = 0.04). Conclusions: Zinc supplementation increased the serum zinc concentration and significantly reduced the ESA dose and ERI, suggesting that a correction of hypozincemia contributes to lessening renal anemia in these patients.

## 1. Introduction

We examined the effects of zinc acetate hydrate supplementation on renal anemia with hypozincemia. Renal anemia is one of the major complications of chronic kidney disease that can affect prognosis. According to the guidelines, aiming for a serum hemoglobin (Hb) level of 10–12 g/dL is recommended [1,2,3]. Erythropoiesis-stimulating agents (ESA) are frequently used to treat renal anemia, but ESA-resistant anemia has become a problem in recent years [4]. The causes of ESA-resistant anemia include blood loss, iron deficiency, carnitine deficiency, and blood disorders. Deficiencies in trace elements, especially zinc, have also been reported as causes [5]. Based on the above, we believe that ESA-resistant anemia should be thoroughly investigated in the treatment of renal anemia, and zinc deficiency should be constantly monitored.

Zinc is a component necessary for the activation of more than 300 types of enzymes. It also plays an important role in cell division and nucleic acid metabolism. Over-the-counter supplements are also available. Zinc has various physiological effects, including an increase in height (children), skin metabolism, reproductive function, skeletal development, taste, maintenance of sensation, mental or behavioral effects, and immune function [5,6].

On the other hand, treatments for zinc deficiency have not progressed sufficiently. Zinc supplementation from dietary intake is possible but is not appropriate for patients with chronic kidney disease (CKD) because it requires a high-protein diet, such as oysters, which causes hypernitrogenemia. Since some gastric drugs contain zinc, they have long been administered for zinc supplementation. However, gastric drugs do not provide sufficient zinc supplementation. Thus, treatment for zinc deficiency has remained problematic. The recent launch of zinc acetate hydrate and its subsequent administration to several patients has been the latest development in the treatment of zinc deficiency. Polaprezinc, an antiulcer drug, has been used as a therapeutic agent for hypozincemia because it contains zinc. However, this is an off-label use. Recently, the use of zinc acetate hydrate, a drug for the treatment of Wilson’s disease, has been extended to treat hypozincemia [7]. Furthermore, zinc acetate hydrate is superior to polaprezinc in increasing serum zinc levels [8].

We report a clinical case series study of how supplementation with zinc acetate hydrate changed ESA requirements in patients with renal anemia associated with zinc deficiency undergoing hemodialysis.

## 2. Results

The changes in the values of parameters measured before and after the administration of zinc acetate hydrate are described in the subsequent paragraphs.

**Figure 1:**
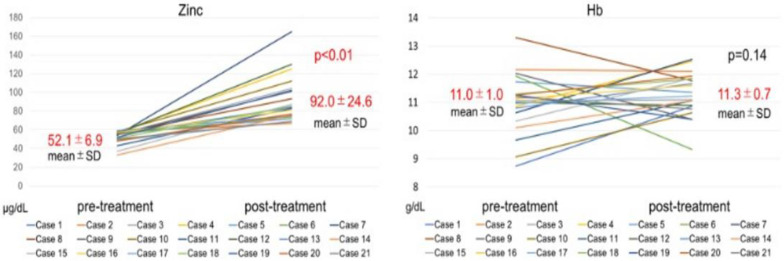
The left side of the figure illustrates the changes in serum zinc levels before and after zinc acetate hydrate administration (for 12 months). The serum zinc level increased significantly after zinc acetate hydrate administration. The right side of the figure reveals the changes in serum hemoglobin (Hb) levels before and after zinc acetate hydrate administration (12 months). Serum Hb levels did not change significantly after zinc acetate hydrate administration. *p*: *p*-value difference; SD: standard deviation.

**Figure 2:**
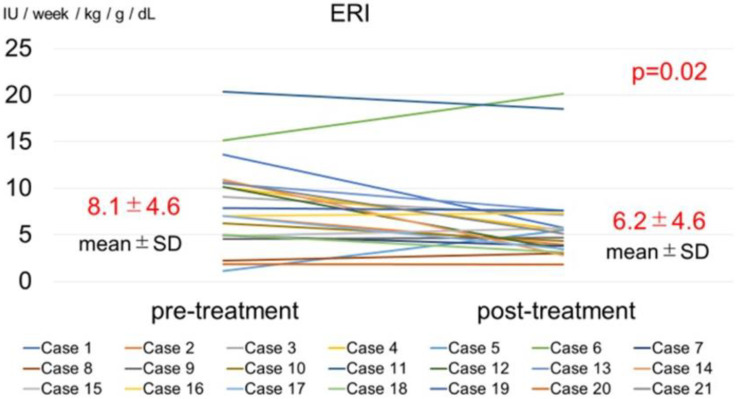
Changes in the erythropoietin resistance index (ERI) before and after zinc acetate hydrate administration (12 months). ERI significantly increased after zinc acetate hydrate administration. *p*: *p*-value difference; SD: standard deviation.

Serum zinc concentration significantly increased, from 33.0–59.0 mg/dL (52.4 ± 7.6 mg/dL) to 57.0–124.0 mg/dL (84.1 ± 16.3 mg/dL; *p* < 0.01) (Figure 1), and blood Hb levels did not change significantly, from 10.0–13.6 g/dL (11.5 ± 1.0 g/dL) to 10.2–12.4 g/dL (11.4 ± 0.7 g/dL) (Figure 1). The ESA dose significantly decreased, from 0–12,000 IU/week (5630 ± 3351 IU/week) to 0–9000 IU/week (4428 ± 2779; *p* = 0.04). The ERI significantly decreased, from 0.0–18.2 (8.1 ± 5.1) to 0.0–16.0 (6.3 ± 4.3; *p* = 0.04) (Figure 2).

**Figure 3:**
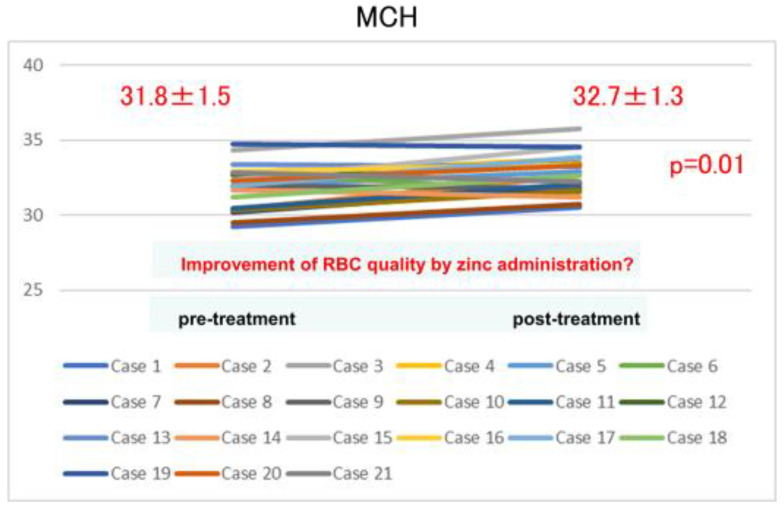
Changes in mean corpuscular hemoglobin (MCH) value before and after zinc acetate hydrate administration (12 months). The MCH value increased significantly after zinc acetate hydrate administration, therefore indicating efficient erythrocyte production. *p*: *p*-value difference; SD: standard deviation.

Figure 3 shows the change in the mean corpuscular hemoglobin (MCH) value before and after zinc acetate hydrate administration (12 months). The MCH value increased significantly after zinc acetate hydrate administration, indicating that erythrocytes were efficiently produced.

**Figure 4:**
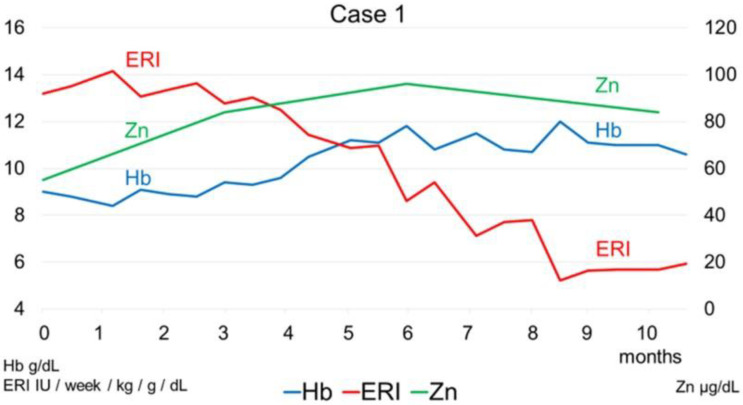
Changes in hemoglobin (Hb), serum zinc, and erythropoietin resistance index (ERI) after zinc acetate hydrate administration in a 54-year-old man who was undergoing hemodialysis therapy due to end-stage renal disease resulting from diabetic nephropathy. The ERI tended to decrease with increasing Hb and serum zinc levels after zinc acetate hydrate administration.

**Figure 5:**
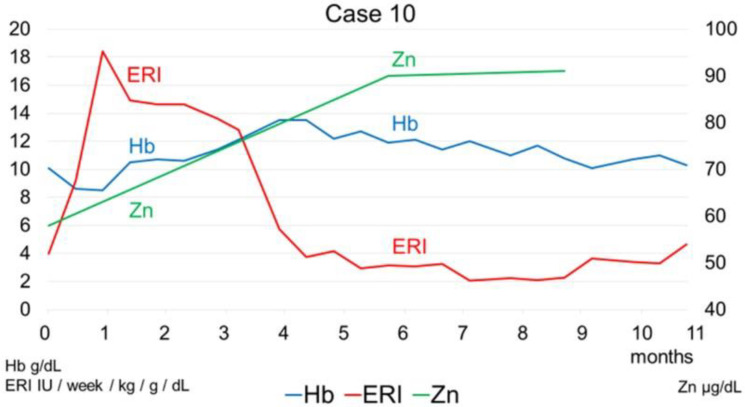
Changes in hemoglobin (Hb), serum zinc, and erythropoietin resistance index (ERI) after zinc acetate hydrate administration in a 62-year-old man who was undergoing hemodialysis therapy due to end-stage renal disease. The ERI value tended to decrease with increasing Hb and serum zinc levels after zinc acetate hydrate administration.

The clinical course of the two cases is shown in Figure 4 and Figure 5. Figure 4 shows changes in Hb, serum zinc, and ERI after zinc acetate hydrate administration in a 54-year-old man who was undergoing hemodialysis due to end-stage renal disease resulting from diabetic nephropathy. After zinc acetate hydrate administration, the ERI tended to decrease with the increase in Hb and serum zinc levels. Figure 5 shows changes in Hb, serum zinc, and ERI after zinc acetate hydrate administration in a 62-year-old man who was undergoing hemodialysis due to end-stage renal disease. After zinc acetate hydrate administration, the ERI also tended to decrease with the increase in Hb and serum zinc levels.

## 3. Discussion

In 1993, Hosokawa et al. reported low serum zinc levels in patients undergoing maintenance dialysis and pointed out the involvement of zinc deficiency in ESA-resistant anemia [9]. However, according to global guidelines on renal anemia and based on the best information available on Hb levels for optimal prognosis, the incidence of ESA-resistant anemia has increased, rendering ESAs ineffective. Deficiencies in trace elements, especially zinc, which is the most abundant element in the living body, have attracted attention. Zinc deficiency affects the activities of various hematopoietic hormones and has been implicated in the mechanism that contributes to anemia. Zinc finger proteins are indispensable for erythroblast differentiation and in the proliferation of GATA-1 [10]. Therefore, zinc deficiency impairs the differentiation and proliferation of erythroblasts [11]. Theoretically, zinc supplementation can be expected to improve renal anemia, but few reports have described the improvement of renal anemia with zinc supplementation.

In a basic experimental study by Feng et al., treatment with zinc compounds in 5/6 nephrectomized rats induced hematopoiesis, similar to recombinant human erythropoietin (rHuEPO) [12]. In a clinical study, Fukushima et al. reported that the administration of a polaprezinc preparation significantly increased serum Hb levels in 58 patients with hypozincemia undergoing hemodialysis [13]. Kobayashi also reported that zinc supplementation reduced ERI in patients undergoing hemodialysis and considered it a novel therapeutic strategy for patients with renal anemia and low serum zinc levels [14]. Although such data are scarce, CKD is not yet fully recognized in clinical practice, especially in patients with hypozincemia associated with renal anemia and undergoing dialysis.

In the present study, we demonstrated that zinc acetate hydrate administration to patients with hypozincemia undergoing hemodialysis produced a significant increase in ERI over the course of 1 year. Perhaps they needed rHuEPO administration; however, more likely, they needed zinc supplementation. Furthermore, in this study, we also found a significant increase in MCH one year after zinc administration, suggesting that the hematopoietic process improved. Regarding the evaluation of MCH, Tomosugi et al. indicated the importance of MCH evaluation in Hb management, in that it leads to the evaluation of the required iron levels for hematopoiesis [15]. Our data do not mention iron requirements, but the significant increase in MCH with zinc may indicate effective hematopoiesis without relying on ESA. No previous report investigating this has been retrieved. It may be regarded as a novel perspective on the relationship between renal anemia and zinc deficiency.

On the other hand, the administration of zinc preparations has undesirable side effects. Of these, hypocupremia is a condition that requires special attention. Munie et al. reported a case involving a patient undergoing hemodialysis who developed hypocupremia and advanced anemia several months after the start of zinc supplementation [16]. Marumo et al. reported that zinc antagonizes copper, so we must take care to diagnose patients ingesting zinc supplements [17]. At our hospital, serum copper concentration is measured every 3 months after zinc acetate hydrate administration. Nevertheless, we have experienced two cases of pancytopenia due to hypocupremia. Of the 21 patients in the present study, none developed hypocupremia. Copper deficiency causes anemia, leukopenia, bone abnormalities, and neuropathy, among other issues. Copper is absorbed through one of two pathways: (1) through the absorption of Cu^2+^ via direct combination with divalent metal transporter 1 and in competition with Fe^2+^ and Zn^2+^, or (2) through a reduction in Cu^1+^ in the duodenum and its subsequent absorption by specifically combining with copper transporter 1, which is present in the brush border membrane of microvilli in the epithelial cells of the small intestine [18,19,20].

In clinical practice, Maruyama et al. found that, out of 816 patients with conservative renal failure, 52.3% had a marginal serum zinc deficiency (60–80 μg/dL), whereas 30.6% had absolute deficiency (<60 μg/dL), indicating that hypozincemia may already be present during the renal failure stage before dialysis [21]. Dhia J. Al-Timimi provided supporting evidence of this, reporting that advancing diabetic nephropathy represented by a decreasing glomerular filtration rate and increasing microalbuminuria was associated with lower serum zinc levels [22]. Thus, determining serum zinc levels and the effectiveness of zinc supplementation in patients with diabetes is crucial, particularly while assessing for kidney damage [14]. Zinc deficiency presents as anemia and has a wide variety of symptoms. Hence, zinc supplementation for zinc deficiency at the stage of non-dialysis kidney disease is desired.

A patient with diabetic nephropathy presented to us with symptoms of zinc deficiency. The patient’s systemic erosive dermatitis improved after the introduction of hemodialysis and the administration of zinc acetate hydrate for 2 weeks. Zinc deficiency also presents other symptoms, such as dysgeusia, which is associated with undernutrition and is considered to be important in the urgent issue of frailty in patients with CKD undergoing dialysis. Therefore, correcting hypozincemia may improve not only renal anemia but also the overall patient condition.

Zinc is one of the most valuable trace elements in the body, and the symptoms caused by zinc deficiency may not be limited to anemia but may also include systemic symptoms, such as hair loss, dermatitis, and dysgeusia. Zinc deficiency is also an important cause of renal anemia in CKD, but reports on this are scarce. However, anemia is very common in CHF. Successful treatment is associated with a significant improvement in cardiac function, functional class, and renal function. Furthermore, a marked fall in the need for diuretics and hospitalization has been noted. Hence, ESA is still used to treat renal anemia in many patients [23], but in reality, ESA requirements are steadily increasing. Problems, such as worsening prognosis due to high-dose ESA preparations, have emerged [24,25,26].

The lack of developments in the treatment of renal anemia may accelerate the vicious cycle of cardiorenal anemia syndrome. Regular monitoring for renal anemia, especially ESA-resistant anemia, is vital. The possibility of renal anemia due to zinc deficiency should be monitored beginning with the stage of conservative renal failure. Renal anemia and malnutrition are important disorders to monitor in patients with conservative renal failure undergoing dialysis. Furthermore, in the patients from this group who develop hypozincemia, the side effects of zinc preparations should be carefully considered.

This examination was impactful because the following two new findings were obtained: First, we used MCH to evaluate the effect of correcting hypozincemia with zinc supplementation on renal anemia. Regarding the evaluation of anemia with MCH, based on our investigation, iron supplementation is recognized. However, zinc supplementation has not been reported. In this study, we were able to show that better-quality hematopoiesis can be performed using easily measurable MCH from the viewpoint of zinc supplementation. Additionally, that zinc acetate hydrate is a zinc preparation with high zinc content. Therefore, it has become possible to provide appropriate zinc supplementation to more hypozincemia patients. Furthermore, we were able to consider the merits and demerits of zinc hydrate preparations. However, further large-scale clinical studies are needed to confirm these findings.

## 4. Conclusions

Zinc acetate hydrate supplementation in patients with zinc deficiency undergoing hemodialysis elicited a significant decrease in ESA requirements. The study findings suggest that appropriate zinc replacement for zinc deficiency is of value in this patient group. The improvement of MCH measurements suggests that the quality of red blood cells (RBC) may have been enhanced by zinc administration.

## 5. Materials and Methods

Our study included 21 outpatients undergoing hemodialysis at Shinmatsudo Central General Hospital, Matsudo city, Chiba prefecture, Japan with serum zinc levels < 60 μg/dL. Patients were excluded if they had hemorrhagic lesions, acute disease (pneumonia, heart failure), hematological disease, or had switched therapies from peritoneal dialysis. We administered zinc acetate hydrate (50 mg per day, reduced to 25 mg per day, as appropriate) to each patient for 12 months. 

We monitored the changes in the ESA low-response index (ERI: ESA dose (IU/week)/dry weight (DW) (kg)/Hb (g/dL)) before and after initiating zinc acetate hydrate treatment. The difference between the two points was evaluated using the Wilcoxon signed rank sum test, and a *p* < 0.05 was considered statistically significant. 

**Figure 6:**
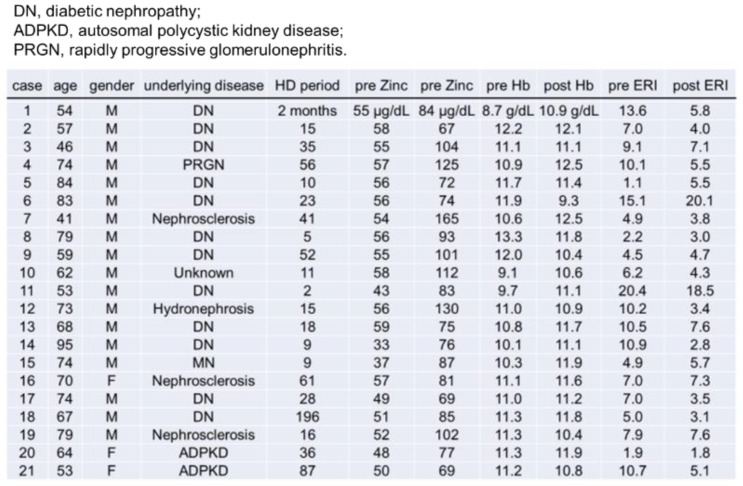
The background of the patients undergoing hemodialysis who received zinc acetate hydrate for renal anemia in this study.

The Figure 6 below presents the background of the 21 patients undergoing hemodialysis who received zinc acetate hydrate for renal anemia in this study.

The study was approved by the Institutional Review Board of Shinmatsudo Central General Hospital (IRB No. 20220003, 8 August 2022). Written informed consent was obtained from all enrolled patients.

## Data Availability

Not applicable.
